# Towards a Program Theory for Family-Focused Practice in Adult Mental Health Care Settings: An International Interview Study With Program Leaders

**DOI:** 10.3389/fpsyt.2021.741225

**Published:** 2021-10-22

**Authors:** Annette Bauer, Stephanie Best, Juliette Malley, Hanna Christiansen, Melinda Goodyear, Ingrid Zechmeister-Koss, Jean Paul

**Affiliations:** ^1^Care Policy and Evaluation Centre, Department of Health Policy, London School of Economics and Political Science, London, United Kingdom; ^2^Australian Institute of Health Innovation, Macquarie University, North Ryde, NWS, Australia; ^3^Department of Psychology, Clinical Child and Adolescent Psychology, Philipps University, Marburg, Germany; ^4^School of Rural Health, Monash University, Melbourne, VIC, Australia; ^5^Austrian Institute for Health Technology Assessment GmbH, Vienna, Austria; ^6^Department of Psychiatry, Psychotherapy and Psychosomatics, Division of Psychiatry I, Medical University Innsbruck, Innsbruck, Austria

**Keywords:** program theory, family-focused practice, evidence-supported practice, implementation, evaluation, parental mental health, theory of change

## Abstract

**Objectives:** In several high-income countries, family-focused practice programs have been introduced in adult mental health care settings to identify and support children whose parents live with mental health problems. Whilst their common goal is to reduce the impact of parental mental illness on children, the mechanisms by which they improve outcomes in different systems and settings are less well known. This kind of knowledge can importantly contribute to ensuring that practice programs achieve pre-defined impacts.

**Methods:** The aim of this study was to develop knowledge about relationships between contextual factors, mechanisms and impact that could inform a program theory for developing, implementing, and evaluating family-focused practice. Principles of a realist evaluation approach and complex system thinking were used to conceptualize the design of semi-structured in-depth interviews with individuals who led the implementation of programs. Seventeen individuals from eight countries participated in the study.

**Results:** Interviewees provided rich accounts of the components that programs should include, contextual factors in which they operated, as well as the behavior changes in practitioners that programs needed to achieve. Together with information from the literature, we developed an initial program theory, which illustrates the interconnectedness between changes that need to co-occur in practitioners, parents, and children, many of which related to a more open communication about parental mental health problems. Stigma, risk-focused and fragmented health systems, and a lack of management commitment were the root causes explaining, for example, why conversations about parents' mental illness did not take place, or not in a way that they could help children. Enabling practitioners to focus on parents' strengths was assumed to trigger changes in knowledge, emotions and behaviors in parents that would subsequently benefit children, by reducing feelings of guilt and improving self-esteem.

**Conclusion:** To our knowledge, this is the first research, which synthesizes knowledge about how family-focused practice programs works in a way that it can inform the design, implementation, and evaluation of programs. Stakeholder, who fund, design, implement or evaluate programs should start co-developing and using program theories like the one presented in this paper to strengthen the design and delivery of family-focused practice.

## Introduction

Family-focused practice approaches, which recognize the family as a unit in the treatment of a person's mental health problems, have been developed and introduced in mental health services internationally. They have in common that they seek to combat the limitations of services that are focused only on the individual, and do not consider the impact of mental disorders on other family members, in particular children (1, 2). Examples of policy initiatives or national flagship programs introducing such approaches include: the ‘Effective Family Program' in Finland (3), ‘Think Family' initiative in the United Kingdom (4), ‘Children of Parents with a Mental Illness' (COPMI; https://www.copmi.net.au) and ‘Families where a Parent has a Mental illness' (5) in Australia. Governments in Scandinavia even made legal amendments to their health and social care acts, that requires practitioners in adult mental health services to identify and look after the needs of children whose parents are using their services (6–8).

The term family-focused practice (FFP) has been used differently in different contexts, and can refer to (mental) health, social care and other sectors. For the purpose of the paper, and in line with Foster et al. (1), we define FFP as the way, by which mental health practitioners or services respond to the family members of the person in treatment for their mental illness. More specifically, we focus on FFP in adult mental health settings and therefore use the term to refer to how adult mental health practitioners and services respond to children.

Even though attempts to transform adult mental health services to incorporate FFP began decades ago (1), most mental health systems still do not operate in this manner (9–11). Reasons for this are manifold, and include fragmented service systems, inadequate funding to address needs beyond the individual's most urgent problems, lack of organizational commitment and leadership reflected, for example, in a lack of policies or guidelines on identifying parenting status, and limited knowledge or skills among practitioners (12, 13).

Whilst the above-mentioned policies and legal changes seek to address barriers, their success depends on efforts to implement changes in local systems and organizations. Such efforts, to change practice at an organizational and local system level, are the subject of our investigation in this paper. We define these efforts as practice change programs, which are complex interventions that require or demand some form of professional behavior change at an individual or collective level (14). From here onwards, we refer to them simply as programs. Most programs have multiple components, which can include the documentation of parenting status, assessment of family relationships and the children's situation and providing or referring to psychological, -educational, -social interventions to support adults in their parenting role or to support children directly (1, 4, 15). Whilst findings from systematic reviews (16, 17) suggest that psychoeducational and psychological interventions can lead to improved mental health for children, evidence is still largely lacking for such multi-component programs that have been implemented under real-world conditions, and which take place in complex ecological systems (18, 19).

In this study, we wanted to understand how the different components of programs have been implemented, and the mechanisms or processes by which they were expected to lead to changes in outcomes for practitioners, parents and children. The goal of our study was to gather knowledge that could inform the development of an initial program theory for FFP. We sought to surface some of the conscious and subconscious processes of how programs have been developed and implemented by interviewing people who had led the implementation of programs in this field. We expected that this kind of explorative knowledge could inform the development of future frameworks that are theory-driven whilst empirically focused, a gap that has been highlighted by various implementation scientists (20–22).

In our understanding of a program theory, we borrowed from two theoretical frameworks developed or commonly used in the public health field - a realist approach and complex systems thinking. Both approaches suggest that interventions cannot be uncoupled from the systems in which they are operating, and interventions need to be developed and evaluated considering contextual factors (23). In public and mental health, a realist approach has been central in shifting the focus of intervention development and evaluation from whether something works to what works, for whom, how, and in which context (24). Whilst a realist approach proposes the development of a theory by linking contextual factors with mechanisms that are expected to lead to desirable outcomes (25–28), a complex systems perspective offers ways to theorize interventions as disruptions to dynamic and complex systems (29, 30). The latter includes the analysis of an intervention's ability to change relationships between key players that make up such systems, displace entrenched practices and transform or redistribute resources (31).

Whilst the realist synthesis guided both the design and analysis of the study, a complex systems thinking perspective, together with insights from behavior change theories, informed mainly the analysis. Following a realist synthesis, we gathered knowledge to understand what works, for whom, in which context and why. In addition, following both, realist and complex systems thinking perspectives, we also wanted to gather knowledge about the role of actors and resources in influencing the interaction between programs and local systems. Combining these aspects, we set out the following research questions: (1) What is the context in which programs take place, and how is it modified? (2) Which program components can be distinguished? (3) What are the expected program outcomes and for whom? (4) What are the assumed mechanisms leading to expected outcomes? Or, in other words, why and how do programs work? (5) What are the resources employed for the delivery of programs?

## Method

### General Approach

Realist approaches suggest several methods for extracting knowledge to inform the development of initial program theories. Reflecting the current state of the evidence base, we initially sought to apply a dual approach, which would have consisted of an initial synthesis of the literature and then interviews with individuals who led the implementation of programs (27, 32). Exploring the international academic and gray literature on FFP, we found detailed descriptions of programs, rich accounts of how they had been implemented, and the challenges (4, 7–11, 33, 34). However, we only identified limited information about expected changes for parents or children and mechanisms or processes leading to those. None of the papers set out or referred to a program theory or explained the rationale for evaluating changes in practitioners' behaviors, and the mechanisms leading to improved child and parent outcomes, a gap that has been highlighted (35). We therefore did not conduct a synthesis of the papers. Instead, we drew from the literature for a description of possible programs components to guide our interviews with program leaders. We also used the information more informally to guide the conduct and interpretation of the findings from qualitative interviews.

### Semi-Structured In-Depth Interviews

We conducted semi-structured in-depth interviews to elicit the perspectives of individuals who had been developing, managing, implementing (and evaluating) FFP programs, and explored their first-hand experiences of driving and implementing practice change in this area.

#### Sampling

A two-stage purposive sampling process, using snowballing principles, was adopted to identify individuals who were leading the implementation of programs. First, we approached a selected group of researchers in the field of FFP. We first contacted a handful of researchers who had been invited as experts to a workshop on the topic of parental mental illness in Austria called Ideas lab, which had been organized by the funder of this research with the aim to conceptualize new research in this area (36). We asked those researchers to recommend other researchers to us, who they thought would know about programs internationally. When contacting those researchers, we also invited them to recommend other researchers. At the end of this snowballing process, we had a group of twenty researchers, all of whom had expertise in FFP as evidenced by their publication record in this area. Next, we asked them to recommend individuals who had been leading the implementation of FFP programs. We did not set out specific criteria as we wanted to allow for diverse programs, including, for example, those that had evolved more organically. Whilst we originally had set out that programs should refer to adult mental health settings, we allowed for the inclusion of programs that spanned across settings or originated from child mental health and social care settings. This decision was made as it became clear from the feedback we received from researchers that the question in which part of the care system the program started or was anchored depended on national or regional funding structures and arrangements. It also became clear that roles of developing, implementing, evaluating, or advocating for programs were overlapping, and that recommended individuals often had more than one role. Often their role was not a formal program administrator role. We therefore did not specify the role or function of individuals should have. Researchers identified altogether forty individuals, who we then invited to participate in the study. Invited participants were from the following countries: Austria, Australia, Germany, Netherlands, Norway, Sweden, United Kingdom, and the US.

#### Study Participants and Data Collection Procedure

We conducted interviews with the 17 individuals who agreed to participate, who were from seven countries (none of the invited participants from Sweden responded to our emails). The rest of the potential participants (*n* = 23) did not respond to our emails. Most interviewees were employed by organizations that provided publicly funded adult or child mental health services. A few were – either additionally or solely – employed by universities or charities or worked in private practice. In addition to clinical and therapist roles, part of their job descriptions covered service improvement, implementation management, or research. Interviewees had professional backgrounds in psychiatry, psychology, occupational therapy, social work, or teaching.

Interviews were conducted via Skype or telephone and, in one instance, in person, as requested by the interviewee. One interview involved two persons from the same program. Interviews lasted between 1.5 and 2 hours. In one case the interview had to be ended earlier than planned, after about thirty min, because the interviewee needed to attend to an emergency concerning a family at their practice. Fully informed verbal consent was obtained at the beginning of each interview, and in writing, which participants completed before or after the interview. The study of interviews was reviewed and approved by the Research Ethics Committee of the London School of Economics and Political Science.

The interview schedule was informed by ideas from realistic synthesis. It included questions about how the program components identified by us from the literature work in practice, the kind of resources their implementation involved (e.g., training), and how they were linked to improved outcomes. We included questions and prompts about ‘how' and ‘why' interviewees thought that outcomes were achieved. This was done to generate knowledge about potential processes and mechanism leading to improved outcomes, and to distinguish between short-, medium- and long-term outcomes. We applied the following order of questions First, we asked interviewees how they had become involved in this area as well as their roles and responsibilities in programs. Next, we asked about their views concerning the key components of programs which we identified from the literature: identifying and documenting parenting status; leading conversations with parents about their parenting and their children; initiating conversations with children about their parent's mental health problems; offering or signposting to interventions and support. We asked them whether they thought some components were more important than others or were more challenging to implement than others. We then asked about the types of outcomes and impacts that they expected from the program, and the processes leading to such outcomes. Finally, interviewees were asked about the context in which the programs took place, the drivers and challenges for change, and the resources and support required to achieve change and overcome challenges. The interview guide is presented in the Supplementary Material.

Interview questions were sent in advance to interviewers, to overcome potential language barriers as not everyone was fluent in English. All but four interviews were conducted in English. The four were conducted in German, which was the preferred language for these interviewees, and the mother language of the lead researcher (AB) who conducted the interviews.

#### Recordings, Translations, and Data Analysis

Audio-recordings were produced for all interviews. Full transcripts of each audio recording were generated and uploaded on NVivo11 software. The coding framework was developed and refined in an iterative process, led by AB and in consultation with members of the research team, with main inputs from a specialist qualitative researcher (JP).The lead researcher (AB) coded the data in NVivo11 following principles of the Framework Method (37), a method that is commonly applied in qualitative health research. JP read a sample of the interviews and provided critical inputs to the development of the coding framework, and into the coding of the data. Main categories of the coding framework reflected the key concepts for developing program theories following a realist synthesis (38): components, context, mechanisms, outcomes, activities, actors, and resources. Data was indexed according to this framework. Within each of the indexed categories, we looked for further themes and created additional (sub-) categories inductively to, allowing, for example, a distinction into practitioners, parent, and child perspectives. Sub-categories were iteratively constructed through conversations between two authors (AB and JP), informed by ideas from behavior change and complex systems theories. Data were then summarized in a matrix by categories using a spreadsheet. For each sub-category a short descriptive summary was generated, which was presented alongside example quotes. In several meetings throughout the study, researchers from the team discussed emerging themes and findings, applying their multi-disciplinary backgrounds in health and social care research to the interpretation of the data.

## Results

We present the findings structured by key concepts. This includes a description of the contextual factors that influence the delivery and outcomes of the program (research question 1), the components of programs, including what those should encompass (research question 2), the expected outcomes for practitioners, parents and children (research question 3), and processes leading to these outcomes (‘mechanisms') (research question 4). Whilst information about resource inputs (research question 5) are provided under the headings of components and contextual factors, we also summarized them briefly in a separate section. At the end of the section, we present an initial program theory that was developed based on these findings.

### Contextual Factors

From interviewees' responses, we identified a range of factors that influenced the successful delivery of programs and outcomes for children. Interviewees described how the stigma, discrimination and social isolation children experienced, often prevented or hindered effective engagement of families with services.

“The degree to which they [families] were avoidant of mental health services because of (…) shame and stigma is massive.” (Interview 8)

“A lot of the children grow up thinking that they are the only child of a parent with a mental illness (…). A lot of these families are isolated or fragmented or stigmatized.” (Interview 9)

Whilst none of the interviewees described a role for programs in changing stigma or awareness at a community level, they emphasized the importance of psychoeducation and helping families to find a language in which they could talk about parental mental illness within the family and to others. (This is described in more detail in the section on mechanisms and outcomes of programs).

Interviewees offered detailed accounts on what had hindered and facilitated practice change at a system and organizational level. They reflected how, traditionally, professional workforce development, education, funding, and performance systems were all focused on the medical treatment of a person's crisis rather than on preventing problems through integrated solutions. Such systems had led to or facilitated certain attitudes, beliefs and behaviors of mental health practitioners, which included them being highly protective of their relationships with ‘their patients'. Most interviewees described what they thought were exaggerated fears among practitioners that if they started to ask detailed questions about parenting and children, this would bring up safeguarding issues, which would require involvement of child welfare agencies and ultimately lead to children's removal from home. Some interviewees reported how they had addressed such barriers by providing accurate information to practitioners about the role of child and youth welfare agencies and safeguarding procedures. This included information about the role of those agencies in supporting families to prevent child removal, and about the risk that children would be removed permanently, which was very small. Some interviewees explained how they had organized cross-sector training with practitioners from adult mental health and child and youth welfare agencies in order to reduce misconceptions that practitioners had about each other's roles.

“So, I have to address that very clearly when talking about this to adult psychiatry personnel that this is not about alerting child protection. That this is the last resort that will be necessary for only a few (…).” (Interview 10)

“(…) there is a lot of misconceptions about child protection services and their work, but I think just reframing it all and saying we want to come into the family as early as possible because there is this possibility of prevention (…).” (Interview 5)

Interviewees described how drivers for successful program delivery had included policies and legislation that were supportive of prevention- and family-focused practice, in particular if those were accompanied with ring-fenced funding for this population. Interviewees explained how their own persuasion or advocacy efforts needed to take place at many different levels in order for change to happen: from policy makers and commissioners of services to senior managers, and frontline practitioners. They described how they had successfully used stories of lived experiences, research data, and legislation on child rights to get the attention of politicians and commissioners. At an organizational level, interviewees referred to the support that managers needed in order to implement changes and the need for organizational capacity to make changes sustainable. This was particularly challenging in organizations that had weak leadership, and in which managers were not skilled to manage organizational change. They described a diverse range of training and workforce development programs that they had implemented. However, according to interviewees training on its own was not sufficient to achieve change in a context, in which frontline practitioners were burnt out and in which there was high staff turnover.

“(…) training works a bit but it doesn't really work to change culture. We have to have lots of things. We have to have the service, the development. You have to have some interventions to help. You have to have the combination. So, it's a whole combination that is needed so that you get that kind of light bulb moment.” (Interview 11)

### Program Components

#### Routine Questions About Parenting Status and Children

Most interviewees explained that, whilst formally and routinely asking parents about their children, and recording this information should be standard practice in adult mental health services, this was commonly not the case. Instead, this was often left to the discretion of the individual practitioner. Recording data on children in the clinical notes (e.g., how many; what age; where they live) was regarded an important starting point for potential further changes in practice. For example, it could lead to sharing information in meetings where case records were reviewed, and to further signposting to support. Some interviewees believed that introducing routine documentation required performance management systems to check compliance.

“We know that parental mental illness has consequences [for children], but we need to find them [the children] in order to help them. So, the idea is to get all the services to systematically ask “Do you have children?”, and to record that, so we can find the children who need help. That has been the main issue, the first step, because we can't provide any family-focused practice if we don't know if the patient has a family.” (Interview 1)

Engaging parents and children, the latter often referred to by the interviewees as “invisible” or “hidden” (terms commonly used in the literature), was described as a major challenge. Therefore, asking the right questions, which could include questions about the wider family network, was regarded as important.

Some interviewees emphasized that practitioners also needed to understand why they were asking those questions, and what they would do with the information.

“In some cases, some of the government policies say you need to ask about children and to find out in which care they are and find out different things. But sometimes people were asking the question, but they didn't have the knowledge and understanding to interpret the information they got back.” (Interview 2)

#### Conversations With Parents About Impact of Mental Health Problems on Children

Interviewees described how discussions between practitioners and parents about the impact of mental illness on their parenting role was a ‘natural' starting point, which could then lead to further conversations about how children were doing, and the impact the parent's mental illness had on them.

“The first conversation, the conversation with the adults is easier for them [adult mental health practitioners], because (…) they already have a relation with the patient.” (Interview 3)

Whilst some interviewees thought that parents were just “waiting for therapists to ask” (Interview 5) about their children, as this was an “existential” part of their identity (Interview 4), others thought that practitioners needed substantial time and efforts to encourage parents to see the benefits of talking with their children about their mental disorder. Some described how motivating the parents to have these discussions could be extremely challenging especially when parents had a limited awareness of their mental illness, which they explained was particularly common among parents with personality disorder. At the same time, interviewees believed that not asking about parenting was potentially harmful, because it reinforced the taboo around the subject.

Interviewees emphasized that conversations needed to follow a strengths-based approach focusing on what the parent was doing well and their needs rather than an assessment of their parenting skills.

A few interviewees also thought that it was important to talk to the wider family as they brought in a different perspective that was not covered by talking to parents or children alone. Since parents with mental disorders often distanced themselves from their wider families, talking to them could help children become less isolated.

#### Conversations With Children About Parental Mental Health

Interviewees described the opportunities for supporting children through adult mental health services. Whilst interviewees agreed that adult mental health services had an important role in facilitating support for children, they had differing views concerning the nature of such involvement. Most interviewees thought that practitioners should encourage parents to have conversations with their children about the impact of their mental health on them. Some interviewees thought that this could or should include talking to children directly.

“I do think quite strongly that adult mental health workers should be able to do that [talking to children]. (…) Because children do slip through the net (…)” (Interview 2)

“Because they know the parent's diagnosis and how this is affecting the parent they [adult mental health practitioners] are the key personnel to explain this to the child.” (Interview 1)

However, other interviewees expressed concerns about practitioners talking to children as this, in their view, required specialist knowledge, skills and dedicated time. Interviewees mentioned some practical barriers in offering help to children in adult mental health settings, such as the need for parental consent, or that some children did not want to talk to professionals involved in their parent's care, as they were worried that something they would say would then be shared with the parent.

“We don't want the adult practitioner of the parent to talk also with the children because for the children, it is important they feel they can talk to someone, who is not connected to the parent.” (Interview 6)

#### Supporting Children, Including in Collaboration With Other Services

Interviewees talked about a wide range of interventions and activities that had been implemented as part of programs to support families and children, ranging from psychosocial and -educational support, to peer support, help with school, leisure and fun activities.

“(…) we came up with [activities] to do with the children… and then, while the children were in class next door, we were educating them [the parents] about child development and about children's experiences of mental illness” (Interview 7)

Whilst some interviewees described informal activities or therapeutic approaches that they had developed themselves in response to what they perceived families needed or wanted (e.g., a fun day, or a support group), others referred to more structured interventions that followed manuals and tools. The latter included genograms for the systematic assessment of social relationships and support needs, evidence-based interventions, such as the Beardslee family intervention (39) and family conferences. Some mentioned a collaboration with researchers in the field, which had informed the development of their support offers and therapeutic methods.

Although some thought there needed to be a specific ‘offer' for this population of children and parents to which practitioners could refer directly, others thought that most communities had existing support offers for families and children in place and that those should be better utilized for these families.

Interviewees believed it was important that adult mental health services collaborated with services and agencies in contact with the family such as child welfare agencies, schools, and mental health services. They thought that the responsibility for supporting this group of children needed to be a shared responsibility between various services. This required a system, in which providing information about mental illness and signposting parents to support was the responsibility of all agencies involved with families. They explained that this required the commitment of all agencies and could only be achieved through wider system changes.

### Program Mechanisms and Outcomes

Interviewees reported on a wide range of behavior changes in practitioners, parents, and children that programs sought to achieve. The following provides description of those, highlighting the connections between outcomes for practitioners, parents, and children as they became apparent to us during the analysis.

#### Practitioners

Interviewees described how practitioners needed to feel confident in talking to parents and motivating them to engage in conversations about parenting and children, as their confidence projected on to the parent. To do this, they also needed to believe in the importance and benefits of doing so and required appropriate skills in delivering strengths-based practice and knowledge about parenting and child development. Whilst changing practitioners' knowledge of the impact of parental mental health problems on children was seen as an important first step by some, others reported that most practitioners knew this but thought that this, on its own, did not lead to changes in practice. In addition to the organizational support structures that needed to be in place, practitioners also needed to experience the impact of parents' mental disorder on children's lives, including the positive impact as a result of their own changes.

“Having information and having knowledge does matter, but what is more important is being able to see the connection between general knowledge and their [parents and children] daily life situations.” (Interview 10)

#### Parents

Interviewees explained that parents needed to understand the importance of talking to their children about their mental health problems as some parents did not think that their mental health problems had an impact on children.

“What is important is that parents realize that they need support and that their children need support.” (Interview 4)

Awareness alone was, however, not always enough, according to interviewees. Parents needed to be willing and able to talk and listen to their children.

“When children ask questions [about parents' mental health problems] it is important, that parents are prepared and that parents are willing to answer questions.” (Interview 12)

Interviewees thought that once families were able to talk openly about parental mental illness, many positive outcomes could be achieved (although they did not further specify which ones, or how they would be achieved), and that this was the change they were focusing on.

“I do think that helping parents and children and other family members to understand what is happening in the family is one of the most important things.” (Interview 13)

“Making this something we can talk about and not making this a big dark secret (…) making them [the parents] able to talk about the problems in their family that's the behavior change we want to achieve.” (Interview 1)

Interviewees explained that by focusing on parents' strengths in their therapy, this would enable them to feel more confident in their parenting skills, and reduce their feelings of shame and guilt, which in turn would improve their mental health symptoms. They described that talking about parenting could lead to improvements in their therapy goals, which in turn changed practitioners' motivation to include family discussions in their therapy.

#### Children

Whilst interviewees were giving comprehensive and coherent accounts of the changes they expected to occur in practitioners and parents, their accounts of changes in children were more diverse. In their reflections on what and how support to children should be provided, the age of children was a main consideration. Interviewees described how discussions with children, initiated by the parent or the practitioner, needed to be conducted using age-appropriate language, and approaches that were focused on the child, their needs, and what mattered to them.

Interviewees described the importance of helping children to understand parents' mental illness, and to enable them to make sense of what was going on at home. Children were feeling relieved once they had more accurate information about their parents' mental illness because they were better able to understand their parents' behaviors and place it outside themselves.

“For the children, the main outcome will be to reduce feelings of guilt and shame (…).” (Interview 1)

A few interviewees described how this new understanding had also improved relationships between children and parents.

“It [talking about parent's mental illness] opened-up a level of trust that had not been there before and it reduced a lot of resentment that had built over the years.” (Interview 9)

Some interviewees thought that these changes led to resilience in the long-term. Other long-term outcomes mentioned by interviewees included improved school performance, prevention of child removal, and reduced trauma (associated with child removal). Some interviewees were convinced that positive long-term prevention effects occurred for children but did not offer an explanation about the types of outcomes, and how those were achieved.

“So, if the parents feel like they are confident and they can do this. They talk to their children about what is going on and it [has] a big prevention effect for the children.” (Interview 1)

Not everyone was certain whether long-term outcomes, such as breaking the cycle of poor mental health between family members, was ultimately achievable, but that it was more about providing children with the tools to cope with adversities. This included children's increased ability to ask for help by helping them to find a language to talk about their parent's mental disorder without shame.

“Obviously we want children, who grow up well, who have resilient lives, and who are able to go on and function well and don't end up with their own mental health issues but (…) [even with support] you could end up with one [mental illness] (…) But [with support] it is more like that - if things go wrong - [the children] are resourceful enough to be able to find, get support and help to work through things.” (Interview 7)

One interviewee reflected on the challenges of evidencing long-term outcomes.

“We are not tracking parents over historic periods, so we are left with relatively short snapshots.” (Interview 9)

### Resources

As mentioned above, a lack of dedicated resources to FFP was seen as a major barrier towards the adoption of FFP. Resource inputs required to implement the program, included different types of training, ongoing supervision, and various opportunities for knowledge exchange between professionals from different agencies, including child and youth welfare, schools, and primary health care. Interviewees considered the commitment from the organization's senior management essential, but explained how a lack of funding for activities that were not core business (together with a lack of change management or general leadership skills) prevented such commitments. Interviewees also talked about commitments required from insurance companies and local, regional, or national governments. Buy-in from these parties were needed to mobilize the necessary resources. Most interviewees thought that, in addition to workforce development, the introduction of new and consistent policies and procedures, which outlined the expectations towards managers and practitioners, as well as (amendments to) reporting and performance systems to monitor those were needed. In addition, interviewees explained that it required a shared vision and care pathways, which needed to be implemented at a system level.

### Initial Program Theory

**Figure 1:**
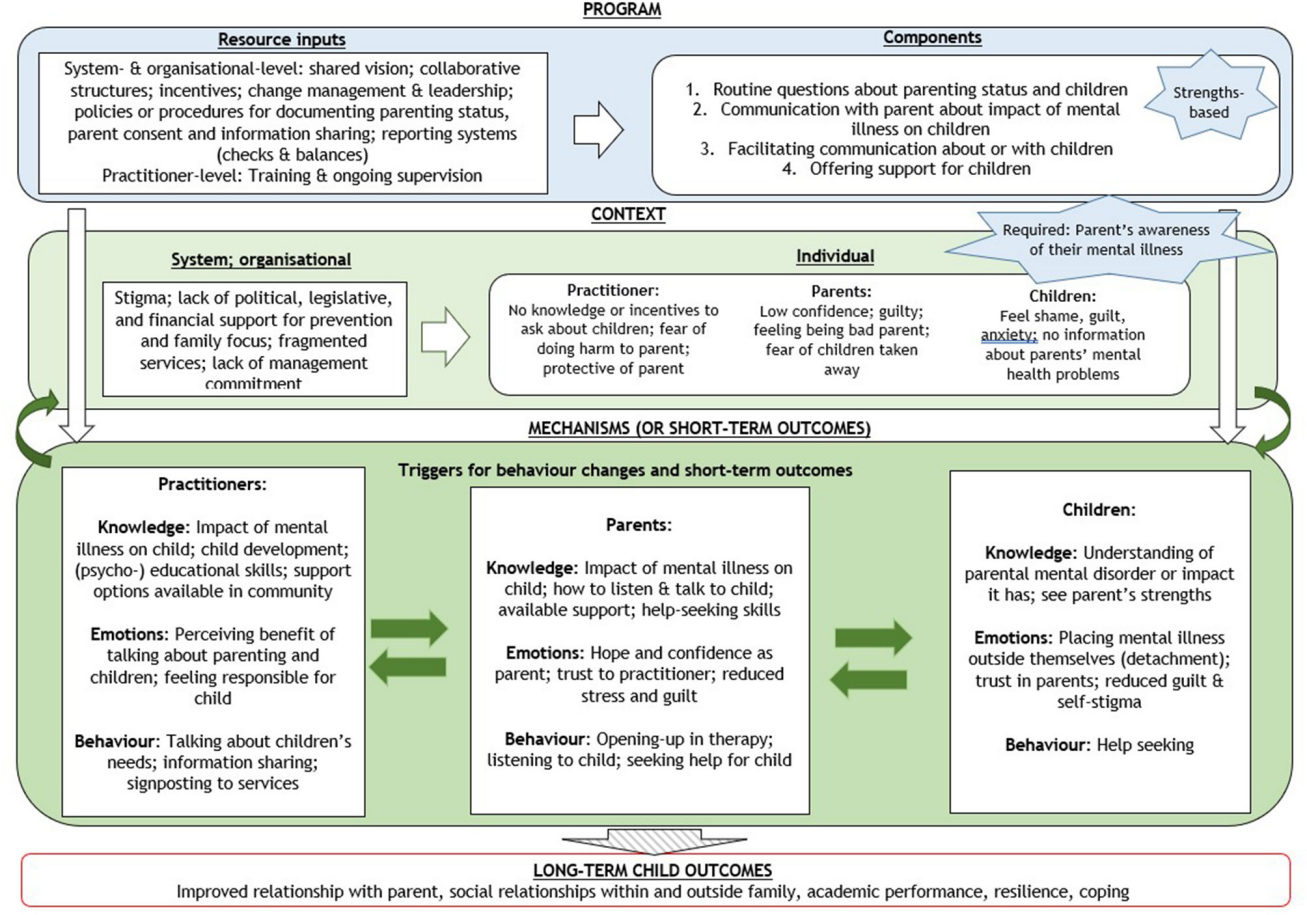
Initial program theory for family-focused practice.

Based on the findings from the interviews, we developed an initial program theory in the form of a logic model, depicted in Figure 1. The logic model illustrates the relationships between resource inputs required to deliver the program components, the contextual factors, which enable or constrain the delivery of the program, and the mechanisms assumed to lead to final long-term outcomes for the child. In the model, we assume that contextual factors are potentially amenable to the programs, and that all or some of them might need to be modified to achieve the desired impact. For example, system and organizational factors, such as stigma, risk-focused and fragmented systems, and lack of management commitment, were assumed to be the root causes of the problem, which impact on practitioners', parents' and children's situations and behaviors, explaining, for example, why they would not have conversations about parents' mental illness. Their knowledge, attitudes, and beliefs, such as those manifested in shame and guilt, present factors at the individual level that need to be addressed by programs.

It is hypothesized that a successful program triggers changes in knowledge, emotions and behavior in practitioners, parents, and children, which are closely interconnected. For example, as practitioners start applying their newly gained knowledge and skills in asking about parenting using a strengths-based approach, they find that parents respond positively, which in turn encourages them to continue with their new practice, and to further enhance their knowledge and skills. As parents are enabled to talk with children about their mental health problems, and learn to listen to the child's needs, children start to develop trust towards their parents, and feel better about themselves. It is expected that this encourages the parent further to talk about their mental illness more openly, both in their relationship with practitioners and their children. More immediate changes in children's feelings and behaviors, such as help-seeking, are then assumed to lead to some longer-term outcomes for children, such as resilience and improved relationships.

Arrows in the Figure 1 illustrate the spiral effects between mechanisms or short-term outcomes for practitioners, parents, and children, as well as possible feedback loops between them and contextual factors. In line with complex system thinking (40), the logic model shows how programs need to activate a virtuous circle where an initial success creates the conditions for further successes. The non-linear way, in which change may be created, was well illustrated by one interviewee:

“[The question is] whether you need to change systems before you can change practitioners before you can change outcomes of the family, or whether you can use changes in families to create changes in practitioners as well. And I used to think they are quite linear (…) but I am less [convinced] by it now and I think that changes in a client can create change in their [practitioners] practice and that enables them to put more things in place organizationally as well.” (Interview 3)

## Discussion

Programs seeking to introduce FFP in adult mental health settings need to be informed by appropriate evidence, which includes evidence about what works in different contexts for different populations, and why it works. This paper contributes to the literature by providing a synthesis of the potential components that constitute FFP programs, and how programs might lead to improvements. To our knowledge, this is the first paper in this field, which synthesizes such knowledge in a way that it can inform the design, implementation, and evaluation of programs. Applying realist and complex system perspectives to our interviews with individuals, who were leading the implementation of programs, allowed us to identify potentially important spiral effects and feedback loops between changes in the behavior of practitioners, parents and children. We were able to identify contextual factors that programs might need to target to trigger such spiral effects. Ultimately, program theories need to be developed for individual programs in collaboration with relevant stakeholders. However, we hope that the knowledge generated in this study provides a useful starting point for such exercises.

Our study was exploratory. Several limitations in our data hindered the development of a more comprehensive and ultimately more robust program theory. A first limitation relates to our main data source, which was a relatively small number of interviews, conducted with interviewees based in a small number of selected countries, all of which were high-income countries. We were not able to reach interviewees from some of the countries in which FFP programs have been implemented, such as Canada, Finland, and Sweden. Whilst selecting a small sample of individuals based on their knowledge and expertise is considered appropriate for the purpose of developing a program theory (41), it might mean that important perspectives from individuals not involved in those networks or movement(s) have been missed. For example, future inquiry is needed to understand whether including a larger number of individuals, including study participants who did not respond to our emails, would validate the initial program theory developed in this study. In addition, we relied in our choice of interviewees on recommendations from expert researchers, and we did not apply clearly defined inclusion criteria to guide their recommendations. It might be that a more refined inclusion of individuals would have led to richer information, such as information about child outcomes. For example, it might be useful to select interviewees by their level of competence and experience in the field, or by certain characteristics of programs they implemented such as size. However, despite this limitation, it was possible to identify commonly held views and common experiences, especially concerning practitioners' attitudes and behaviors, and how those needed to be changed. Whilst it was beyond the remit of this study to include the views of service users' representatives, future research should involve families using services.

Overall, in our data we observed that information was much richer for the short-term outcomes of programs, which is not uncommon in program theories as many interventions only seek to achieve intermediate outcomes (42). However, program theories should be transparent about which outcomes they seek to achieve, how short-term or intermediate outcomes are linked to long-term outcomes (if at all) and highlight evidence gaps. For example, the focus on short-term outcomes might be indicative of an insufficient evidence-base for child-focused practice and of an uncertainty about what kind of outcomes can be expected for children of different age groups (35, 43). It also is possible that, especially in some adult mental health settings, where the focus is naturally on the adult, the final outcomes of FFP are perceived to be about achieving parent's outcomes [potentially alongside children's outcomes). In addition, other outcomes such as those for partners or siblings, might be considered important too. A program theory should make the expectations as to what are viewed as final outcomes clear, and set out the pathways that are supported by evidence and can be realistically assumed to be causal vs. those that are less well established (42). For FFP, future enquiry is needed to assess which types of evidence should be considered when developing the initial program theory further.

The findings from our study also highlight the importance of including the expected relationships between behavior changes in practitioners, parents, and children into program theories, and how those (in combination) influence longer-term child outcomes. For example, the role of trusting, non-judgmental relationships between practitioners and families have been found to lead to improved parents' mental health (35), and good interpersonal relationships between children and their parents have been found to lead to improved child behavior (19). Additional actors might be useful to include, such as individuals managing, funding, or influencing FFP. Integrating theories of behavior change, which describe the dynamic relationships between players at different organizational or system levels, into program theories might be particularly valuable. Methods that support the development of this knowledge, such as actor-based change framework (44), social network analysis (31, 45) and the Capability, Opportunity, Motivation towards Behavior change approach (COM-B) (46) might be particularly useful for developing context-sensitive strategies as part of practice change programs (47).

Another area that program theories should address (but commonly do not) refers to economic evidence. Economic evidence in FFP is largely lacking (35). Whilst we identified cost pressures as a key barrier that prevented change in this area, a finding that is commonly cited in the relevant literature (12), only a few interviewees mentioned the importance of developing an economic case for programs in this area. Program theories, in particular if they include economic evidence, can be an important tool to address accountability demands of funders and tax payers in systems that are under financial pressure (44). They can also be an important tool to help building a collation for change in systems in which many stakeholders from different organizations and sectors are involved, and which require democratic processes to agree on common goals and actions to achieve those.

Different types of program theories may be developed using a range of methodologies, for example supporting the specific purpose of each of the stages of the program development, implementation, and evaluation cycle (40, 47). An initial program theory, such as the one we developed, might play a particular important role during the early development stage, which benefits particularly from theories that consider the interactions of the program with contextual factors (47). Developers might first set out the contextual factors that are most pertinent to the successful delivery of their program, the components they want to focus on as a result, and describe those in detail, together with the resource inputs they require. In the case of FFP this might include discussions about: whether and how mental illness stigma needs to be addressed through the program, whether reporting and performance systems are fit for purpose, how managers might need to be supported to lead change processes. Without such planning, it is possible that programs fail. For example, introducing new staff roles in adult mental health settings to take on additional responsibilities to look after children largely failed in the context of the strongly hierarchical Swedish and Norwegian systems, in which important decisions are traditionally only made by doctors (48).

Actions to prevent child and youth mental health problems are expected to lead to long-lasting improvements in wellbeing, health, and employment (49). Considering that one in four to five children live with parents with mental health problems (50) and that the risk for those children to develop mental health problems is as high as forty per cent (51), use of evidence-based practice in this area is important. Our paper provides a starting point for an increased use of program theories in this important area of practice.

## Data Availability Statement

The raw data supporting the conclusions of this article will be made available by the authors, without undue reservation.

## Ethics Statement

The studies involving human participants were reviewed and approved by London School of Economics and Political Science. The patients/participants provided their written informed consent to participate in this study.

## Author Contributions

AB was primarily responsible for the study design and conduct and analysis as well as manuscript preparation for this study. JP also informed the study design and supported the conduct and analysis of the study. All authors contributed to the conceptualization of the study, final analysis and preparation of the manuscript, and approved the submitted version.

## Funding

The research described in this paper was conducted as part of the research project How to raise a village to raise a child, which received funding from the Austrian Federal Ministry of Health, Science and Research through the Open Innovation in Science Centre at the Ludwig Boltzmann Gesellschaft GmbH. The project is hosted by the Medical University of Innsbruck, which provided in kind contributions to the research project. The funders did not influence the collection, analysis, and interpretation of data and played no role in writing the manuscript.

## Conflict of Interest

The authors declare that the research was conducted in the absence of any commercial or financial relationships that could be construed as a potential conflict of interest.

## Publisher's Note

All claims expressed in this article are solely those of the authors and do not necessarily represent those of their affiliated organizations, or those of the publisher, the editors and the reviewers. Any product that may be evaluated in this article, or claim that may be made by its manufacturer, is not guaranteed or endorsed by the publisher.

## References

[1] Foster K Maybery D Reupert A Gladstone B Grant A Ruud T. Family-focused practice in mental health care: an integrative review. Child and Youth Services. (2016) 37:129–55. 10.1080/0145935X.2016.1104048

[2] Nicholson J Reupert A Grant A Lees R Maybery D Mordoch E. The policy context and change for families living with parental mental illness. Parental Psychiatr Diso. (2015). 3:354–64. 10.1017/CBO9781107707559.034

[3] Solantaus T Toikka S. The effective family program: preventative services for the children of mentally ill parents in finland. Int J Mental Health Promot. (2006) 8:37–44. 10.1080/14623730.2006.9721744

[4] Grant A Lagson S Devaney J Davidson G Duffy J Perra O. A study of health and social care professionals' family focused practice with parents who have mental illness, their children and families in Northern Ireland. Final report. Retrieved from Belfast, Ireland. (2018).

[5] Goodyear M Hill T-L Allchin B McCormick F Hine R Cuff R. Standards of practice for the adult mental health workforce: meeting the needs of families where a parent has a mental illness. Int J Ment Health Nurs. (2015) 24:169–80. 10.1111/inm.12120 25619407

[6] Afzelius M Plantin L Östman M. Families living with parental mental illness and their experiences of family interventions. J Psychiatr Ment Health Nurs. (2018) 25:69–77. 10.1111/jpm.12433 28906576

[7] Lauritzen C Reedtz C Van Doesum K Martinussen M. Factors that may facilitate or hinder a family-focus in the treatment of parents with a mental illness. J Child Fam Stud. (2015) 24:864–71. 10.1007/s10826-013-9895-y 25814823 PMC4363479

[8] Reedtz C Lauritzen C Stover YV Freili JL Rognmo K. Identification of children of parents with mental illness: a necessity to provide relevant support. Front Psychiat. (2019) 9:728. 10.3389/fpsyt.2018.00728 30670987 PMC6333019

[9] Goodyear M Maybery D Reupert A Allchin R Fraser C Fernbacher S. Thinking families: A study of the characteristics of the workforce that delivers family-focussed practice. Int J Ment Health Nurs. (2017) 26:238–48. 10.1111/inm.12293 28026142

[10] Maybery D Foster K Goodyear M Grant A Tungpunkom P Skokoy BE. How can we make the psychiatric workforce more family focused? Parental Psychiatr Diso. (2015) 3:301–11. 10.1017/CBO9781107707559.029

[11] Maybery D Goodyear M O'Hanlon B Cuff R Reupert A. Profession differences in family focused practice in the adult mental health system. Fam Process. (2014) 53:608–17. 10.1111/famp.12082 24945363

[12] Maybery D Reupert A. Parental mental illness: a review of barriers and issues for working with families and children. J Psychiatr Ment Health Nurs. (2009) 16:784–91. 10.1111/j.1365-2850.2009.01456.x 19824972

[13] Shah-Anwar S Gumley A Hunter S. Mental health professionals' perspectives of family-focused practice across child and adult mental health settings: a qualitative synthesis. Child Youth Services. (2019) 40:383–404. 10.1080/0145935X.2019.1591947

[14] Johnson MJ May CR. Promoting professional behavior change in healthcare: what interventions work, and why? A theory-led overview of systematic reviews. BMJ Open. (2015) 5:e008592. 10.1136/bmjopen-2015-008592 26423853 PMC4593167

[15] Liangas G Falkov A. Use of structured clinical documentation to identify patients' parental concerns and their childrens' wellbeing. Commun Ment Health J. (2014) 50:646–55. 10.1007/s10597-013-9684-5 24532226

[16] Siegenthaler E Munder T Egger M. Effect of preventive interventions in mentally ill parents on the mental health of the offspring: systematic review and meta-analysis. J Am Acad Child Adolesc Psychiatry. (2012) 51:8–17.e18. 10.1016/j.jaac.2011.10.018 22176935

[17] Thanhäuser M Lemmer G de Girolamo G Christiansen H. Do preventive interventions for children of mentally ill parents work? Results of a systematic review and meta-analysis. Curr Opin Psychiat. (2017) 30:283–99. 10.1097/YCO.0000000000000342 28505032

[18] Nicholson J. Guest editorial. Aust e-J Adv Mental Health. (2009) 8:222–6. 10.5172/jamh.8.3.222

[19] Solantaus T Paavonen EJ Toikka S Punamäki R-L. Preventive interventions in families with parental depression: children's psychosocial symptoms and prosocial behavior. Eur Child Adolesc Psychiat. (2010) 19:883–92. 10.1007/s00787-010-0135-3 20890622 PMC2988995

[20] Greenhalgh T Papoutsi C. Studying complexity in health services research: desperately seeking an overdue paradigm shift. BMC Med. (2018) 16:95. 10.1186/s12916-018-1089-4 29921272 PMC6009054

[21] Lawless A Baum F Delany-Crowe T MacDougall C Williams C McDermott D. Developing a framework for a program theory-based approach to evaluating policy processes and outcomes: health in all policies in South Australia. Int J Health Policy Manag. (2018) 7:510–21. 10.15171/ijhpm.2017.121 29935128 PMC6015512

[22] Reed JE Howe C Doyle C Bell D. Simple rules for evidence translation in complex systems: a qualitative study. BMC Med. (2018) 16:92. 10.1186/s12916-018-1076-9 29921274 PMC6009041

[23] Craig P Di Ruggiero E Frohlich K Mykhalovskiy E White M on behalf of the Canadian Institutes of Health Research (CIHR) National Institute for Health Research (NIHR) Context Guidance Authors Group. Taking account of context in population health intervention research: guidance for producers, users and funders of research. Retrieved from Southampton: NIHR Evaluation, Trials and Studies Coordinating Centre. (2018). 10.3310/CIHR-NIHR-01

[24] Duncan C Weich S Fenton S-J Twigg L Moon G Madan J. A realist approach to the evaluation of complex mental health interventions. The British Journal of Psychiatry. (2018) 213:451–3. 10.1192/bjp.2018.96 30027875

[25] Blamey A Mackenzie M. Theories of change and realistic evaluation: peas in a pod or apples and oranges? Evaluation. (2007) 13:439–55. 10.1177/135638900708212933389043

[26] Byng R Norman I Redfern S. Using realistic evaluation to evaluate a practice-level intervention to improve primary healthcare for patients with long-term mental illness. Evaluation. (2005) 11:69–93. 10.1177/1356389005053198

[27] Jackson SF Kolla G. A new realistic evaluation analysis method: linked coding of context, mechanism, and outcome relationships. Am J Evaluat. (2012) 33:339–49. 10.1177/1098214012440030

[28] Pawson R. Nothing as practical as a good theory. Evaluation. (2003) 9:471–90. 10.1177/1356389003094007

[29] Moore GF Evans RE. What theory, for whom and in which context? Reflections on the application of theory in the development and evaluation of complex population health interventions. SSM - Population Health. (2017) 3:132–5. 10.1016/j.ssmph.2016.12.005 29302610 PMC5742639

[30] Moore GF Evans RE Hawkins J Littlecott H Melendez-Torres GJ Bonell C. From complex social interventions to interventions in complex social systems: Future directions and unresolved questions for intervention development and evaluation. Evaluation. (2018) 25:23–45. 10.1177/1356389018803219 30705608 PMC6330692

[31] Hawe P Shiell A Riley T. Theorising interventions as events in systems. Am J Community Psychol. (2009) 43:267–76. 10.1007/s10464-009-9229-9 19390961

[32] Mukumbang FC van Belle S Marchal B van Wyk B. Towards developing an initial program theory: program designers and managers assumptions on the antiretroviral treatment adherence club program in primary health care facilities in the metropolitan area of western cape province, South Africa. PLoS ONE. (2016) 11:e0161790–e0161790. 10.1371/journal.pone.0161790 27560352 PMC4999218

[33] Korhonen T Vehviläinen-Julkunen K Pietilä AM. Implementing child-focused family nursing into routine adult psychiatric practice: hindering factors evaluated by nurses. J Clin Nurs. (2008) 17:499–508. 10.1111/j.1365-2702.2007.02008.x 18205682

[34] Tchernegovski P Reupert A Maybery D. Let's Talk about Children: A pilot evaluation of an e-learning resource for mental health clinicians. Clin Psychol. (2015) 19:49–58. 10.1111/cp.12050

[35] Bee P Bower P Byford S Churchill R Calam R Stallard P. The clinical effectiveness, cost-effectiveness and acceptability of community-based interventions aimed at improving or maintaining quality of life in children of parents with serious mental illness: a systematic review. Health Technol Assessment. (2014) 18:1–250. 10.3310/hta18080 24502767 PMC4780907

[36] Ludwig Boltzmann Gesellschaft (2021). Ideas Lab. Available online at: https://ois.lbg.ac.at/en/projects/ideas-lab. (accessed September 13, 2021)

[37] Ritchie J Lewis J. Qualitative Research Practice: A Guide for Social Science Students and Researchers. London: Sage. (2003).

[38] Pawson R Tilley N. Realistic Evaluation (Vol. 2). London: SAGE Publications. (1997).

[39] Beardslee WR Gladstone TR Wright EJ Cooper AB. A family-based approach to the prevention of depressive symptoms in children at risk: evidence of parental and child change. Pediatrics. (2003) 112:e119–131. 10.1542/peds.112.2.e119 12897317

[40] Rogers PJ. Using program theory to evaluate complicated and complex aspects of interventions. Evaluation. (2008) 14:29–48. 10.1177/1356389007084674

[41] Manzano A. The craft of interviewing in realist evaluation. Evaluation. (2016) 22:342–60. 10.1177/1356389016638615

[42] Wight D Wimbush E Jepson R Doi L. Six steps in quality intervention development (6SQuID). J Epidemiol Commun Health. (2015) 70:520–5. 10.1136/jech-2015-205952 26573236 PMC4853546

[43] Caldwell DM Davies SR Hetrick SE Palmer JC Caro P López-López JA. School-based interventions to prevent anxiety and depression in children and young people: a systematic review and network meta-analysis. Lancet Psychiat. (2019) 6:1011–20. 10.1016/S2215-0366(19)30403-1 31734106 PMC7029281

[44] Koleros A Mulkerne S Oldenbeuving M Stein D. The actor-based change framework: a pragmatic approach to developing program theory for interventions in complex systems. Am J Eval. (2018) 41:34–53. 10.1177/1098214018786462

[45] Shelton RC Lee M Brotzman LE Crookes DM Jandorf L Erwin D. Use of social network analysis in the development, dissemination, implementation, and sustainability of health behavior interventions for adults: a systematic review. Soc Sci Med. (2019) 220:81–101. 10.1016/j.socscimed.2018.10.013 30412922 PMC7857673

[46] Michie S van Stralen MM West R. The behavior change wheel: a new method for characterising and designing behavior change interventions. Implement Sci. (2011) 6:42. 10.1186/1748-5908-6-42 21513547 PMC3096582

[47] Mills T Lawton R Sheard L. Advancing complexity science in healthcare research: the logic of logic models. BMC Med Res Methodol. (2019) 19:55. 10.1186/s12874-019-0701-4 30871474 PMC6419426

[48] Östman M Afzelius M. Children's representatives in psychiatric services: what is the outcome? Int J Soc Psychiat. (2009) 57:144–52. 10.1177/0020764008100605 19875625

[49] Knapp M Evans-Lacko S. Health economics. In Rutter's Child and Adolescent Psychiatry. (2015). p. 227–238. 10.1002/9781118381953.ch18

[50] Abel KM Hope H Swift E Parisi R Ashcroft DM Kosidou K. Prevalence of maternal mental illness among children and adolescents in the UK between 2005 and 2017: a national retrospective cohort analysis. Lancet Public Health. (2019) 4:e291–300. 10.1016/S2468-2667(19)30059-3 31155222 PMC6557735

[51] Foster K. One-third of children of parents with severe mental illness are at risk of developing severe mental illness. Evid Based Mental Health. (2014) 17:73–73. 10.1136/eb-2014-101807 24994669 PMC13404257

