# Supplementary material for: Towards a Program Theory for Family-Focused Practice in Adult Mental Health Care Settings: An International Interview Study With Program Leaders
Source: Front Psychiatry. 2021 Oct 22;12:741225. doi: 10.3389/fpsyt.2021.741225 (PMC8568767; doi:10.3389/fpsyt.2021.741225)
Supplement: Supplementary file 1 [file Data_Sheet_1.docx]

*Supplement*

Topic guide for interviews with expert practitioners who led the implementation of family-focused practice programmes in adult mental health services

SECTION 1: INTRODUCTION

- **Thank the person for participating in the research**
- **Provide the person with the aims of the discussions and a brief outline of the research** (they will have had information from the invite so this can be brief)
- **Explain briefly about yourself** (for example taking role of naïve interviewer or expert from another field)
- **Remind person of informed consent** (usually, the interviewee will have read and signed informed consent form – if not yet signed, explain process and ask person for verbal consent ; inform them that we will approach them for written consent afterwards; this includes information about data recording and their right to withdraw from the research at any time)
- **Explain the interview process to person** (i.e. the number of questions and overall duration 45 minutes; what will happen after the interview; when the recording starts and ends)
- **Provide participant with an opportunity to ask questions about the research and interview process**
- **Inform them you will start recording now**

**TURN ON RECORDER**

SECTION 2: QUESTIONS

DISCUSSION 1: About them (their role and experience)

Explain to participant that you would like to find out more from them about their current and past experience in working to bring a family perspective to mental health services.

1. Briefly, could you tell me a bit about HOW and WHY you became involved in working in this area?
2. How would you summarise your current and past roles and responsibilities to bring about practice change in this area?
3. What would you say were the key objectives of the practice changes you implemented (or plan to implement), and HOW were they achieved (or, if in planning stages, how can they be achieved)?

*If you were involved in different approaches, could you focus on the key objectives of the one that you think worked best and tell us why you think it worked best?*

Suggested prompts: Why do you think that people wanted to do this practice change? What did they think they could achieve and how? What were peoples’ expectation of it?

1. Would you say that supporting practitioners or other professionals was an important part of your role? If so, could you explain why it was important?
2. What was the nature of your role in supporting practitioners or other professionals?

Explain to participant that based on our initial literature review we identified different areas and components that practitioners or other professionals might need support with, including:

- Recording and reviewing information about parenting status and children (e.g. number, age, living with parent vs. in care)
- Talking with adults about their *parenting role and identity, and the impact being a parent and having a family has on their illness*
- Talking with adults who are parents about the *impact of their illness on their children*
- Talking with adults who are parents about *seeking support for their child*
- Talking with children about their *parent’s mental illness*
- Talking with children about their and their *own support needs*
- Sharing information with others such as practitioners and professionals who interact with families, and collaborating more widely

1. Would you agree that this list of components is useful when thinking about workforce support? Could you tell us why do you think it is useful (not useful)? Are there any components we missed?
2. Do you think that some components are more important to work towards in order to bring a family perspective to mental health services for adults that are parents? If so, WHY?
3. From your experience, are some components more challenging to implement than others? If so, WHY?

DISCUSSION 2: Outcomes and mechanisms

Explain to participant that we would now like to talk to them about their thoughts on how bringing a family perspective to adult mental health services can change outcomes for families.

1. From your perspective, what were/ are the most important outcomes for *parents*? Could you explain your choice? WHAT do you think made/ can make those outcomes happen?
2. From your perspective, what were/are the most important outcomes *for children*? Could you explain your choice? WHAT do you think made/ can make those outcomes happen?
3. Would you say that the impact was / is for some children or parents greater than for others? If so, which groups of children or parents are likely to benefit more (less), and WHY?
4. Do you think that in order for some of those impacts to happen, certain changes need to be achieved first? If so, which ones and WHY?
5. What do you believe is the single, most important practice change that needs to occur in adult mental health settings in order to generate positive outcomes for children or their families?
6. In your opinion, what could be an important indicator of success in achieving the desired practice change?

DISCUSSION 3: Context

Explain to participant that you would like to talk to them a bit about the national and local context in which they work/ worked to achieve practice change. Explain that by context we mean: Legislation and policy; financial climate; funding models; service infrastructure; communities’ characteristics; organisational culture; management and work conditions; supervision and mentoring; practitioners’ relationships; relationships between practitioners and families; families’ relationships.

1. In terms of the above topics, HOW would you describe the context, in which you implemented the practice change?
2. WHY (and HOW) did the context you just mentioned matter?
3. What kind of challenges did you and other practitioners or professionals, who were driving practice change, face and HOW did you or they overcome those challenges (if at all)?
4. In terms of the (organisational or management) support you received (if any), what would you say helped you the most, and WHY?
5. If you could choose one type of support that you think would help you the most in driving practice change, what would that be, and WHY?

DISCUSSION 4: Finalise discussion by asking questions about their professional role. Ask them to state which ONE position describes their own best.

Clinician

- Clinician in adult mental health (inpatient)
- Clinician in adult mental health (outpatient; community)
- Clinician in adult mental health (inpatient) with management responsibility
- Clinician in adult mental health (outpatient, community) with management responsibility
- Other clinician – please specify

Researcher

- Clinical researcher in adult mental health
- Clinical researcher in child mental health
- Non-clinical researcher in adult mental health
- Non-clinical researcher in child mental health

Manager or coordinator

- Non-clinical manager (or coordinator) in adult mental health (inpatient)
- Non-clinical manager (or coordinator) in adult mental health (outpatient, community)
- Non-clinical manager (or coordinator) in child mental health (inpatient)
- Non-clinical manager (or coordinator) in child mental health (outpatient, community)
- Other manager – please specify

Positions with specific service development/ practice change responsibilities

- Liaison between adult mental health and child services
- Other liaison – please specify
- Implementation specialist
- Service development manager
- None of the above – please specify

Other

- Please specify

End the interview by thanking the interviewee for participating in the interview.
